# Development of an Ion Sensitive Field Effect Transistor Based Urea Biosensor with Solid State Reference Systems

**DOI:** 10.3390/s100606115

**Published:** 2010-06-21

**Authors:** Kow-Ming Chang, Chih-Tien Chang, Kun-Mou Chan

**Affiliations:** Department of Electronics Engineering, Institute of Electronics, National Chiao Tung University, Hsinchu, 30050, Taiwan; E-Mails: kmchang@cc.nctu.edu.tw (K.M.C.); daily.deli@msa.hinet.net (K.M.C.)

**Keywords:** ion sensitive field-effect transistors (ISFETs), reference field-effect transistors (REFETs), differential measurement, biosensor, enzyme field-effect transistors (EnFETs), transconductance-match

## Abstract

Ion sensitive field-effect transistor (ISFET) based urease biosensors with solid state reference systems for single-ended and two-ended differential readout electronics were investigated. The sensing membranes of the biosensors were fabricated with urease immobilized in a conducting polymer-based matrix. The responses of 12.9∼198.1 mV for the urea concentrations of 8∼240 mg/dL reveal that the activity of the enzyme was not significantly decreased. Biosensors combined with solid state reference systems were fabricated, and the evaluation results demonstrated the feasibility of miniaturization. For the differential system, the optimal transconductance match for biosensor and reference field-effect transistors (REFET) pair was determined through the modification of the membranes of the REFETs and enzyme field-effect transistors (EnFETs). The results show that the transconductance curve of polymer based REFET can match with that of the EnFET by adjusting the photoresist/Nafion™ ratio. The match of the transconductance curves for the differential pairs provides a wide dynamic operating measurement range. Accordingly, the miniaturized quasi-reference electrode (QRE)/REFET/EnFET combination with differential arrangement achieved similar urea response curves as those measured by a conventional large sized discrete sensor.

## 1. Introduction

Field-effect transistor (FET) based solid state biosensors are a promising tool in biological applications due to the maturity of semiconductor technology. In particular, the feasibility of miniaturization enables advanced applications, such as the surgical operations [1]. Enzyme field-effect transistors (EnFETs) are miniaturized biosensors which are typical ion-sensitive FET (ISFET) based biosensors, with an additional immobilized enzyme layer coating on the surface of gate dielectrics of a FET [2–6]. Those additional layers were fabricated on the supporting materials with entrapped enzyme coatings. Most biological molecules such as enzymes, receptors, antibodies, cells *etc.* have very short lifetimes in solution phase and thus they have to be fixed in a suitable matrix. The immobilization of the biological component to protect it from the environmental conditions results in decreased enzyme activity [7]. The activity of immobilized molecules depends upon surface area, porosity, hydrophilic character of the immobilizing matrix, reaction conditions and the methodology chosen for immobilization. Recently, conducting polymers were regarded as suitable candidates as supporting matrix for biological immobilization applications due to their numerous advantages [8].

Nafion™ is one of the popular materials selected as a supporting matrix for chemical or biological sensors [9–11]. It is a perfluorosulfonated material, with high conductivity in the 10^−1^∼10^−2^ S/cm range [12], which has three parts: a hydrophobic fluorocarbon C–F backbone, an interfacial region of relatively large fractional void volume and the clustered regions where the majority of the ionic exchange sites, counter ions, and absorbed water exist. The rigid backbone is resistant to chemical attack, which protects the entrapped materials, such as polymers and enzymes, from dissolving in electrolyte. Meanwhile, the large fractional void volume and conducting property avoid severe degradation of the activity of immobilized molecules.

According to the ISFET sensors with single structure [13] and ISFET/REFET differential pair structure [14], they show that the readout circuits and stable reference systems are essential for miniaturizing purposes. In general, there are two combinations to achieve the purpose : one is the solid-state reference electrode (SRE) with an ISFET (SRE/ISFET) associated with a single ended readout circuit [15,16]; the other one is the noble metal electrode, which is called a quasi-reference electrode (QRE), integrated with an ISFET and a REFET (QRE/ISFET/REFET) associated with two ended differential readout circuits [17–19].

For the first single-ended combination, the design of the readout circuits is simple if SRE provides a stable potential. Extensive developments for the miniaturized solid state reference electrode were proposed [20–24]. However, in order to achieve a thermodynamically defined potential difference at the reference electrode/liquid interface, the complications of the structures are enormous and there are many drawbacks, such as the leakage of the reference solutions that limits the device lifetime and measurement accuracy [25,26].

**Figure 1.**
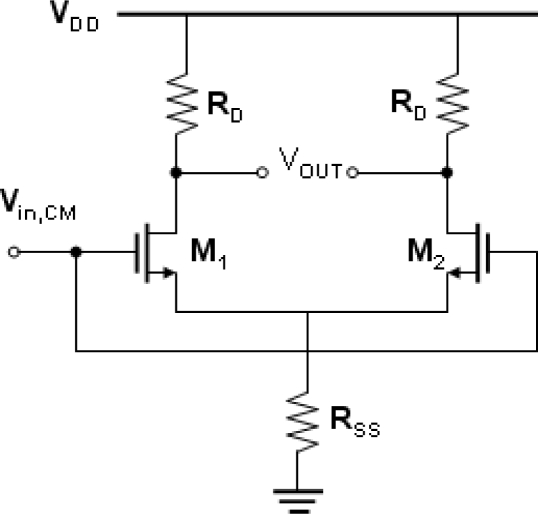
The schematic diagram of the typical common-mode differential circuit.

For the second two-ended combination, the reference electrodes were substituted with the QRE. The QRE is a noble metal which is deposited by a sputtering or evaporation method. Because of the simplicity of the QRE fabrication processes, miniaturized sensors can therefore be achieved easily. However, the designs of the differential readout circuits are more complicated due to the concern of the common mode noise and the ISFET/REFET device match. The common mode noise, induced from the polarized thermodynamically undefined metal/liquid interface, can usually be eliminated using differential methods. Figure 1 shows the schematic diagram of the typical common-mode differential circuit. The purpose of a differential amplifier is to sense the change in its differential input while rejecting changes in its common-mode input. The desired output is differential, and its variation should be proportional to the variation in the differential input. Variation in the common-mode output is undesired. The common-mode rejection ratio (CMRR) of a differential circuit measures the tendency of the device to reject the common signals for both input leads, and it indicates that the amount of the common-mode signal will appear in the measurement. The CMRR was defined as:

(1) $$\mathrm{CMRR}=\left|\frac{A_{\mathrm{DM}}}{A_{\mathrm{CM}-\mathrm{DM}}}\right|$$

where *A_DM_* denotes the circuit gain in the differential-mode, and *A_CM-DM_* denotes the common-mode to differential-mode conversion. An important device parameter regarding the CMRR is the transconductance (g_m_), which represents the sensitivity of the device. For a high g_m_, a small change in V_GS_ results in a large change in I_DS_ at fixed V_DS_, which provides higher measurement sensitivity. The transconductance is defined as in Equation (2):

(2) $$g_{m}=\frac{{\partial I}_{\mathrm{DS}}}{{\partial V}_{\mathrm{GS}}}{|}_{V_{\mathrm{DS}}=\mathrm{cons}\;\text{tan}\;t}$$

Assuming that the g_m1_ and g_m2_ are the transconductances of M1 and M2, respectively. We have:

(3) $$A_{\mathrm{CM}-\mathrm{DM}}=-\frac{{\Delta g}_{m}R_{D}}{(g_{m1}+g_{m2})R_{\mathrm{SS}}+1}$$

where Δ*g_m_* = *g*_*m*1_ − *g*_*m*2_.

According to equations (1–3), a transconductance mismatch of the ISFET/REFET pair will significantly degrade the CMRR, or in other words, the noise coming the from reference electrode metal/liquid interface will influence the measurement accuracy. An alternative method to increase the CMRR is to design high A_DM_ circuits; however, this requires a large number of additional components and increases the complexity of the circuit. Therefore, a transconductance matched ISFET/REFET pair will avoid the costs caused by a degraded CMRR and increase the measurement accuracy.

In this work, three sensor design steps were performed: firstly, the fabrication and characterization of the sole SRE, REFET and ureasable-EnFET. Then, the modifications of transconductance match for devices, and the last step was to evaluate the performances of the sensors with single-ended and two-ended differential combinations based on both the glass Ag/AgCl reference electrode (GRE) and QRE. Nafion™ was used as the common supporting matrix to immobilize functional materials—urease and photoresist—to fabricate the EnFETs, SREs and REFETs. The urea response, response time, storage time and the electrical properties of the sensors were also investigated.

## 2. Experimental Section

### 2.1. Reagent Preparation

Five (5) wt% Nafion™ solutions were obtained from DuPont, and pH buffer solutions were purchased from RDH (Frankfurt, Germany). Photoresist (FH6400) was obtained from Nano Facility Center, National Chiao Tung University. The urease (EC 3.5.1.5, 5 U/mg, lyophilized) was purchased from Merck. Urea [CO(NH_2_)_2_, Merck] and all the other reagents were of analytical grade. The phosphate buffer solution (PBS) was prepared with deionized water. The tested urea solutions were prepared by mixing urea power with PBS, and their concentrations were 1.25, 10, 40, 80, 120 and 240 mg/dL, respectively.

### 2.2. ISFET Fabrication and Membrane Preparation

**Figure 2.**
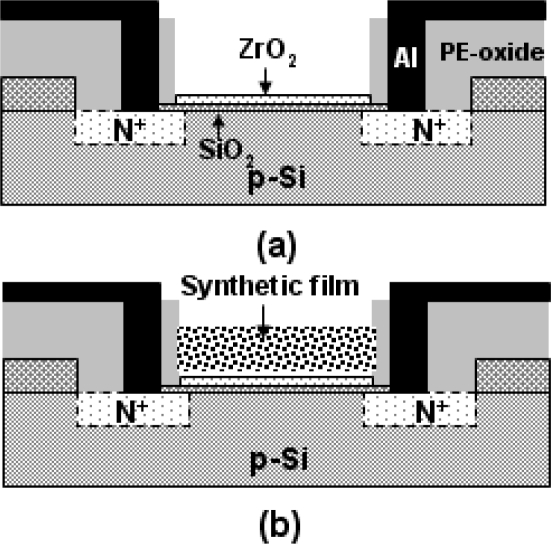
Schematic diagrams of (a) ISFET (b) EnFET and REFET.

The ZrO_2_ gated ISFETs were fabricated by the MOSFET technique. A 30-nm-thickness ZrO_2_ film was deposited onto the SiO_2_ gate ISFET by DC sputtering. The total sputtering pressure was 20 mTorr in a gas mixture of Ar and O_2_ for 200 minutes, while the base pressure was 3 × 10^−^^6^ Torr, and the RF power was 200 W. The quasi-reference electrode (QRE) was fabricated with Ti/Pd deposition by sputtering with a thickness of 150 Ǻ/350 Ǻ. The detailed process flows and characteristics of sensitivity, linearity and drift of the ZrO_2_ ISFET were reported in [27]. The ZrO_2_ gate ISFET exhibited a high pH sensitivity of 57.5 mV/pH. The membranes of solid-state reference electrodes (SREs), and EnFETs were fabricated with the photoresist and urease entrapped in a Nafion™ supporting matrix. Figure 2 shows the schematic diagrams of the ISFET, EnFET and REFET.

In the case of SRE fabrication, the photoresist was mixed with Nafion™ in a 1:1 ratio, and then drop coated on the top of the QRE and dried in air for 24 hours. A similar process was used to fabricate the REFET by drop coating the mixture on the top of the ISFETs; three photoresist/Nafion™ ratios of 1:1, 3:1 and 5:1 were prepared for the REFETs’ test. For the EnFET, the enzymatic layers were prepared by mixing urease solution (10 mg of the urease in 100 μL of 5 mM PBS) with Nafion™ solution (100 μL Nafion™ in 100 μL of 5 mM PBS) in the ratios of 1:1, 5:1 and 20:1, then depositing them on the top of the gate region of the ISFETs by the drop coating method and drying in air for 24 hours. To obtain a consistent membrane thickness, care was taken to control the droplet volume.

### 2.3. Packaging and Measurements

**Figure 3.**
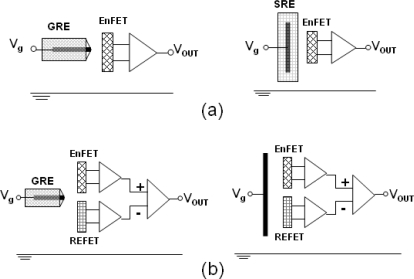
Test structures: (a) single-ended (b) two-ended differential pairs.

Figure 3 shows the combinations of the EnFET measuring systems. A container to enclose the gate region of the ENFET is bonded using epoxy resin. A HP4156A semiconductor parameter analyzer was used to investigate and collect the electrical data. The I_DS_-V_GS_ curves of the EnFETs were obtained with a constant drain-source voltage V_DS_ = 2 V while the devices were soaked in a 10 mM PBS buffer solution of pH = 6. Since the products of the urea hydrolysis may alkalinize maximally up to pH 9, the set initial pH value should cover the range of optimal urease activity (pH 7.0∼7.5). Meanwhile, according to the report in [3], a higher initial pH value of the buffer results in reduction of amplitude of the analytical signal, so accordingly, the initial pH in this experiment was set at pH 6.0. As standard reference electrode, a commercial Ag/AgCl glass reference electrode was connected to the gate voltage supplier to provide the stable bias potential for device operation. In order to prevent any influence caused by light, the measurements were conducted in a dark box. The devices were stored dry at 4 °C in darkness during the measurements.

## 3. Results and Discussion

**Figure 4.**
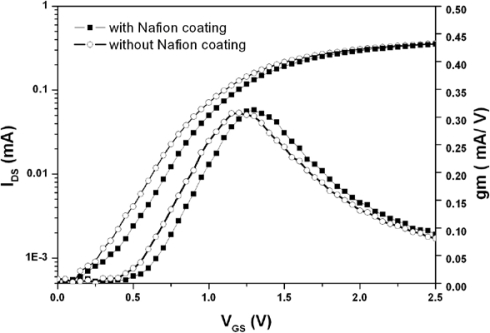
The I_DS_-V_GS_ and transconductance (gm) curves of ZrO_2_ gate ISFETs with and without Nafion™ coating.

Figure 4 shows the I_DS_-V_GS_ and the transconductance curves of the ZrO_2_ gate ISFETs with and without Nafion™ coating. The result reveals that their curvatures are identical, which describes the electrical properties of the Nafion™. The I-V and g_m_ relationships of the ISFETs operating in linear region can be described as follows:

(4) $$I_{\mathrm{DS}}=\frac{1}{2}C_{\mathrm{ov}}\;\mu_{}\left(\frac{W}{L}\right)\left[\left(V_{\mathrm{GS}}-V_{\mathrm{th}}\right)V_{\mathrm{DS}}-\frac{1}{2}V_{\mathrm{DS}}^{2}\right]$$

(5) $$g_{m}=\frac{{\partial I}_{\mathrm{DS}}}{{\partial V}_{\mathrm{GS}}}|{}_{V_{\mathrm{DS}}=\mathrm{cons}\;\text{tan}\;t}=\frac{1}{2}C_{\mathrm{ov}}\;\mu_{}\left(\frac{W}{L}\right)V_{\mathrm{DS}}$$

where *C_OV_* represents the overall capacitance of subsequent layers on sensing area, the μ represents the electron mobility of device and (W/L) represents the geometric ratio of gate. According to equations (4) and (5), since the devices were fabricated with the same size, material and process, the similar curvatures represent the fact that the additional Nafion™ membrane did not alter the overall capacitance of the gate layers.

**Figure 5.**
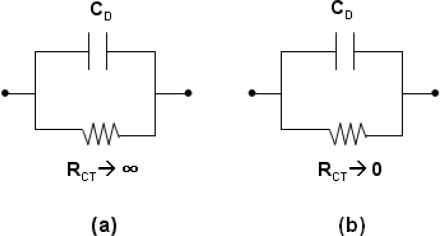
Equivalent circuit of (a) ideally polarizable interface and (b) ideally nonpolarizable interface.

A way to visualize the electrical properties of a membrane is to make use of the simple equivalent circuit representation as shown in Figure 5 [28]. The R_CT_ denotes the charge-transfer resistor and C_D_ denotes the double-layer and membrane capacitor. If the resistor is very high, the capacitor charges up to the value of the potential difference set by the source.

This is the behavior of a polarizable (ion blocking) interface. To polarize an interface means to alter the potential difference across it. On the contrary, if the resistance in parallel with the capacitor is low, then any attempt to change the potential difference across the capacitor is compensated by charge leaking through the low-resistance path. This is the behavior of a nonpolarizable (ion unblocking) interface. With high conductivity and low impact on overall capacitance, the Nafion™ membrane is ion-unblocking and suitable as a supporting material

**Figure 6.**
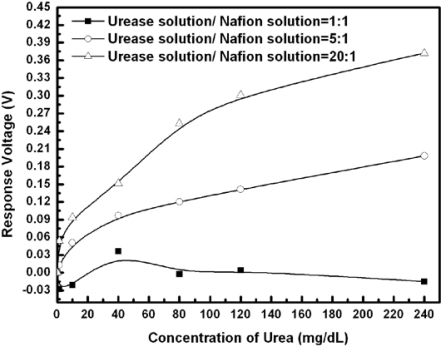
The urea responses of urease-EnFET with different ratio of urease entrapped dL in axis label.

Figure 6 and Table 1 show the responses and performance of the fabricated urease-EnFET measured with standard Ag/AgCl GRE. The concentration of the phosphate buffer solution was 10 mM and the tested urea concentrations were from 1.25 mg/dL to 240 mg/dL. The response performance depends not only on the amount of entrapped urease, but also on the initial pH, ambient buffer capacity and ionic strength of solutions as well as on the surface area, porosity and the physical characteristics of both the enzyme and the supporting material.

The results show that the urea sensitivity was proportional to the amount of urease entrapped in the membrane. Less urease entrapped in the membrane will degrade the urea detection ability. On the other hand, though the sensitivity and detection limits were enhanced with high enzyme loading, the device lifetime was limited due to the enzyme leakage. The entrapment process or membrane confinement of enzyme may be a purely physical caging or involve covalent binding. To enhance the chemical binding capability may increase the enzyme loading and achieve higher sensitivity. In addition to the consideration of the volumetric surface area available to the enzyme, which determines the maximum binding capacity, another consideration was that the nature of supporting material could have a considerable affect on an enzyme’s expressed activity and apparent kinetics. In this experiment, the sensor with the urease/Nafion ratio of 5:1 successfully performed the detection, and the urea responses were from 12.9 mV to 198.1 mV for urea concentrations from 8 mg/dL to 240 mg/dL. It demonstrated acceptable detection ability, response time and life time.

The urease biosensor based on the pH-ISFET detects pH change around the gate surface as a result of the urease catalyses the hydrolysis of urea according to the reaction:

(6) $${\left({\mathrm{NH}}_{2}\right)}_{2}\mathrm{CO}+{3H}_{2}O\overset{\mathrm{urease}}{\to }{\mathrm{HCO}}_{3}^{-}+{2\mathrm{NH}}_{4}^{+}+{\mathrm{OH}}^{-}$$

According to [29], the mechanisms involved in the response of pH-based enzyme sensors include the reaction kinetics of the biological-recognition processes and the mass transport. In the steady state, a balance between the rates of mass transport of the urea from bulk solution to the urease membrane, production or consumption of the hydrogen ions by the urease membrane and their transportation will be achieved, leading to a stable local pH change in the region of the membrane. We can then expect a change in the surface concentration of H^+^ as a consequence of the change in the potential of the ISFET, and the concentration of urea is therefore measured indirectly. On the other hand, biosensors with indirect measurement are also impacted by the ambient buffer capacity and ionic strength, which are governed by many factors such as the concentration, dissociation constant and the ionic charge of electrolyte. For example, the increase of the phosphate buffer solution concentration will result in reduction of the EnFET sensitivity and change the linear part of the calibration curve, as reported in [3,30].

**Figure 7.**
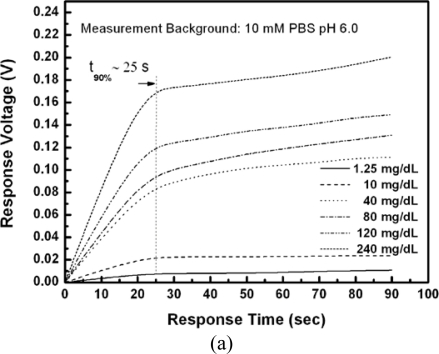

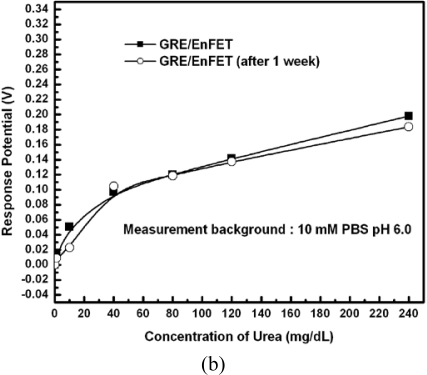
(a) Urea response of the GRE/EnFET (b) Storage and repeatability performances of the GRE/EnFET dL in axis label.

Figure 7(a) shows that the response times of all tests were within 25 seconds. The response time of EnFETs depends on the diffusion of hydrogen ions, the buffer capacity of system and the membrane properties and thickness.

In this experiment, the membranes were fabricated by the drop coating method, and the thickness of membranes were estimated to be 15 μm but not standardized. The membranes were thick; however, the response times were short for all tests, which represented the porosity and ion unblocking property of the membranes with Nafion™ as supporting matrix. Compared with the typical response time of 0.5∼3 minutes. [30], this result demonstrated the quick response characteristics of the proposed EnFET. The storage and repeatability performances of the proposed EnFETs are shown in Figure 7(b) and Table 2.

**Figure 8.**
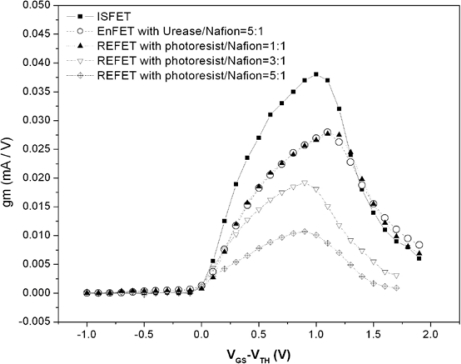
The g_m_ curves of ISFET, EnFET and REFETs.

The results show that the urease entrapped membranes have good repeatability and long storage time. Figure 8 shows the electrical curves measured with GRE. The maximal g_m_ of the ISFET and EnFET were 38 and 27.9 mA/V, respectively. For REFETs, they were 27.7, 19.2 and 10.7 mA/V for the different photoresist/Nafion™ ratios of 1:1, 3:1 and 5:1. Among the devices, the EnFET and REFET curves with the photoresist/Nafion™ ratio of 1:1 were matched. In principle, the transconductance is mainly governed by the electron mobility, gate geometry and effective dielectric capacitance of the sensing layer. In this experiment, all devices were constructed on identical ISFETs, therefore their electron mobility and gate geometry were identical. Accordingly, the different g_m_ curves changed due to the capacitances change caused by the additional membrane. Since both types of membrane involved a similar concept of combining conductive and insulated materials to achieve nonpolarizable interfaces, hence it is possible to modify the overall conductance and capacitance to make FETs electrically match by adjusting their compositions and thickness, *etc.*

The result is particularly important for biosensors with differential readout designs. Most biosensors immobilize biomaterials with chemical, physical or mixed approaches, and the sensing membranes fabricated in each way certainly alter the original electrical properties. The fabrication of REFETs was the similar case. Transconductance mismatched input pairs could induce many issues, such as the CMRR degradation, DC offsets restrict linear dynamic ranges, low voltage gain and nonlinear problems, *etc.* To solve those issues, extra components and complicated designs of circuits and device geometries are essential, therefore increasing the complexity of the readout electronics. Nevertheless, not all the problems induced by mismatch can be overcome. In many proposed novel differential readout circuit designs, the identical electrical properties of the bio-FETs/REFETs pair were assumed [18,31]; in other words, the previous device designs for electrical match can reduce the loadings for circuit designs. Consequently, the g_m_ match for devices design must be considered. The curves in Figures 8 shows the results of designed REFETs with photoresist/Nafion™ ratios of 1:1, 3:1 and 5:1, respectively. It shows that curve of REFETs with photoresist/Nafion™ ratio of 1:1 can match the EnFET curve within the range of V_GS_-V_TH_ = 0∼1.3 V, which provides wide ranges and larger g_m_ choices for readout circuits design considerations. In contrast, the other EnFET/REFET differential pairs only matched at V_GS_-V_TH_ = 0∼0.2 V, in the so-called weak inversion region. As a result, the device sensitivity was relatively low and the dynamic operating range was restricted.

**Figure 9.**
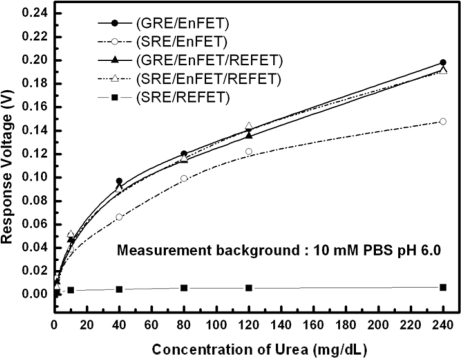
Urea response of test structures of the GRE/EnFET, SRE/EnFET, GRE/EnFET/REFET, QRE/EnFET/REFET and SRE/REFET dL in axis label.

Figure 9 shows the urea responses of GRE/EnFET, SRE/EnFET, GRE/EnFET/REFET, QRE/EnFET/REFET and SRE/REFET combinations in this experiment. The zero urea response of SRE/REFET demonstrated its capability of being a biosensor reference system. Since the REFETs covered with nonpolarized membrane still possesses limited urea sensitivity of around 0.01 mV/mg/dL, the GRE/EnFET/REFET and GRE/EnFET have similar performance, except that the urea sensitivity was slightly lower for the two-ended pair. However, it demonstrated that the fabricated REFETs were practical for use as a reference system. On the other hand, the SRE/EnFET has the simplest on-chip structure with simple one-ended readout circuits. In this work, an altenative method was proposed to fabricate reference electrodes by coating ion-insensitive but ion-unblocking layers on the top of contact metal. This method suppressed the solid/liquid potential differences and provided a relatively stable reference potential. Nevertheless, the result reveals one main disadvantage in that the sensitivity was severely degraded. Comparing the combinations, the on-chip QRE/EnFET/REFET transconductance-match pair with two-ended differential readout circuits demonstrated comparable performance with GRE/EnFET and GRE/EnFET/REFET. Meanwhile, their storage time was more than 1 week. The results indicate the miniaturization of practical ISFET based biosensors can be realized.

## 4. Conclusions

The designed transconductance-match biosensors with solid state reference systems for the differential readout electronics were investigated. Utilizing Nafion™ as supporting matrix provided the advantage of low initial charge-transfer resistance. The entrapment of the ion-blocking materials, such as urease and photoresist, can therefore be performed without altering the unpolarizable property of sensing membrane for EnFETs and REFETs. Meanwhile, through the modification of the membranes of REFETs and EnFETs, the optimal transconductance match for biosensor and REFET pairs can be determined. Such pairs can be easily integrated on-chip due to the simple readout electronics required, and are capable of obtaining biological responses comparable to those of conventional large sized discrete sensors.

## Figures and Tables

**Table 1.** The urea responses and performance of sole urease biosensors with different ratio of urease entrapped.

**Operation Temperature: 25 ° C**					
**Urease solution : Nafion solution**	**Detection Limit (mg/dL)**	**Sensing Range (mg/dL)**	**Sensitivity (mV/per mg/dL)**	**Lifetime**	**Response Time (sec)**

1:1	Not available	Not available	Not available	Not available	Not available
5:1	8	8∼240	0.64	>7 days	25∼60
20:1	1.25	1.25∼240	1.33	<30 min	Not available

**Table 2.** The storage and repeatability performances of GRE/EnFET.

**Stored at 4 °C in darkness**			
**Urea Concentration (mg/dL)**	**Sensor Response (mV)**	**Sensor Response (after 1 week) (mV)**	**Storage Stability (% of sensor response)**

1.25	12.9	8.8	68%
10	50.7	23	45%
40	97.1	104.6	92%
80	120.3	118.7	99%
120	141.4	137.6	97%
240	1988.1	183.6	93%

## References

[1] Covington A.K., Valdes-Perezgasga F., Weeks P.A., Brown A.H. (1993). pH ISFETs for intramyocardial pH monitoring in man. Analusis.

[2] Osaka T., Komaba S., Seyama M., Tanabe K. (1996). High-sensitivity urea sensor based on the composite film of electroinactive polypyrrole with polyion complex. Sens. Actuat. B-Chem.

[3] Pijanowska D.G., Torbicz W. (1997). pH-ISFET based urea biosensor. Sens. Actuat. B–Chem.

[4] Puig-Lleixa C., Jimenez C., Alonso J., Bartroli J. (1999). Polyurethane-acrylate photocurable polymeric membrane for ion-sensitive field-effect transistor based urea biosensors. Anal. Chimica Acta.

[5] Soldatkin A.P., Montoriol J., Sant W., Martelet C., Jaffrezic-Renault N. (2003). A novel urea sensitive biosensor with extended dynamic range based on recombinant urease and ISFETs. Biosens. Bioelectron.

[6] Jaffrezic-Renault N., Wan K., Senillou A., Chovelon J.M., Martelet C. (1999). Development of new polymeric membranes for ENFETs for biomedical and environmental applications. Analusis.

[7] Evtugyn G.A., Budnikov H.C., Nikolskava E.B. (1998). Sensitivity and selectivity of electrochemical enzyme sensors for inhibitor determination. Talanta.

[8] Gerard M., Chaubey A., Malhotra B.D. (2002). Application of conducting polymers to biosensors. Biosens. Bioelectron.

[9] Kinlen P.J., Heider J.E., Hubbard D.E. (1994). A solid-state pH sensor based on a Nafion-coated iridium oxide indicator electrode and a polymer-based silver chloride reference electrode. Sens. Actuat. B.

[10] Volotovsky V., Nam Y.J., Kim N. (1997). Urease-based biosensor for mercuric ions determination. Sens. Actuat. B.

[11] Chang K.M., Chang C.T., Chao K.Y., Chen J.L. (2010). Development of FET-Type Reference Electrodes for pH-ISFET Applications. J. Electrochem. Soc.

[12] Sone Y., Ekdunge P., Simonsson D. (1996). Proton Conductivity of Nafion 117 as Measured by a Four-Electrode AC Impedance Method. J. Electrochem. Soc.

[13] Bergveld P. (1970). Development of an ion-sensitive solid-state device for neuro-physiological measurements. IEEE Trans. Biomed. BME.

[14] Janata J., Huber R.J. (1979). Ion-sensitive field effect transistors. Ion-Sel. Electrode Rev.

[15] Ravezzi L., Conci P. (1998). ISFET sensor coupled with CMOS read-out circuit microsystem. Electron. Lett.

[16] Khanna V.K., Kumar A., Jain Y.K., Ahmad S. (2006). Design and development of a novel high-transconductance pH-ISFET (ion-sensitive field-effect transistor)-based glucose biosensor. Int. J. Electron.

[17] Rocher V., Chovelon J.M., Jaffrezic-Renault N., Cros Y., Birot D. (1994). An Oxynitride ISFET Modified for Working in a Differential Mode for pH Detection. J. Electrochem. Soc.

[18] Ravezzi L., Stoppa D., Corra M., Soncini G., Dalla Betta F., Lorenzelli L. (2001). A CMOS ASIC for differential read-out of ISFET sensors. Electron. Circuits. Syst.

[19] Hammond P.A., Ali D., Cumming D.R.S. (2004). Design of a Single-Chip pH sensor using a conventional 0.6-μm CMOS process. IEEE Sens. J.

[20] Suzuki H., Hirakawa T., Sasaki S., Karube I. (1998). Micromachined liquid-junction Ag/AgCl reference electrode. Sens. Actuat. B.

[21] Shimada K., Yano M., Shibatani K., Komoto Y., Esashi M., Matsuo T. (1980). Application of catheter-tip ISFET for continuous *in-vivo* measurement. Med. Biol. Eng. Comput.

[22] Smith R.L., Scott D.C. A solid state miniature reference electrode.

[23] Huang I.Y., Huang R.S. (2002). Fabrication and characterization of a new planar solid-state reference electrode for ISFET sensors. Thin Solid Films.

[24] Maminska R., Dybko A., Wr′oblewski W. (2006). All-solid-state miniaturised planar reference electrodes based on ionic liquids. Sens. Actuat. B.

[25] Suzuki H., Hiratsuka A., Sasaki S., Karube I. (1998). Problems associated with the thin-film Ag/AgCl reference electrode and a novel structure with improved durability. Sens. Actuat. B.

[26] Suzuki H., Ozawa H., Sasaki S., Karube I. (1998). A novel thin-film Ag/AgCl anode structure for microfabricated Clark-type oxygen electrodes. Sens. Actuat. B.

[27] Chang K.M., Chao K.Y., Chou T.W., Chang C.T. (2007). Characteristics of Zirconium Oxide Gate Ion-Sensitive Field-Effect Transistors. Jpn. J. Appl. Phys.

[28] Grattarola M., Massobrio G. (1998). Bioelectronics Handbook: MOSFETs, Biosensors, and Neurons.

[29] van der Schoot B.H., Bergveld P. (1988). ISFET based enzyme sensors. Biosensors.

[30] Boubriak O.A., Soldatkin A.P., Starodub N.F., Sandrovsky A.K., El’skaya A.K. (1995). Determination of urea in blood serum by a urease biosensor based on an ion-sensitive field-effect transistor. Sens. Actuat. B.

[31] Morgenshtein A., Sudakov-Boreysha L., Dinnar U., Jakoson C.G., Nemirovsky Y. (2004). CMOS readout circuitry for ISFET microsystems. Sens. Actuat. B.

